# The Ups and Downs of Tannins as Inhibitors of Poly(ADP-Ribose)glycohydrolase

**DOI:** 10.3390/molecules16021854

**Published:** 2011-02-22

**Authors:** Christian Blenn, Philippe Wyrsch, Felix R. Althaus

**Affiliations:** Institute of Pharmacology and Toxicology, University of Zurich-Vetsuisse, Winterthurerstrasse 260, 8057 Zurich, Switzerland; E-Mails: philippe.wyrsch@vetpharm.uzh.ch (P.W.); felix.althaus@vetpharm.uzh.ch (F.R.A.)

**Keywords:** tannin, gallotannin, tannic acid, PARG, poly(ADP-ribose)

## Abstract

DNA damage to cells activates nuclear poly(ADP-ribose)polymerases (PARPs) and the poly(ADP-ribose) (PAR) synthesized is rapidly cleaved into ADP-ribose (ADPR) by PAR glycohydrolase (PARG) action. Naturally appearing tannin-like molecules have been implicated in specific inhibition of the PARG enzyme. This review deals with the *in vitro* and *in vivo* effects of tannins on PAR metabolism and their downstream actions in DNA damage signaling.

## 1. The Impact of Poly(ADP-Ribose) Metabolism

**Figure 1:**
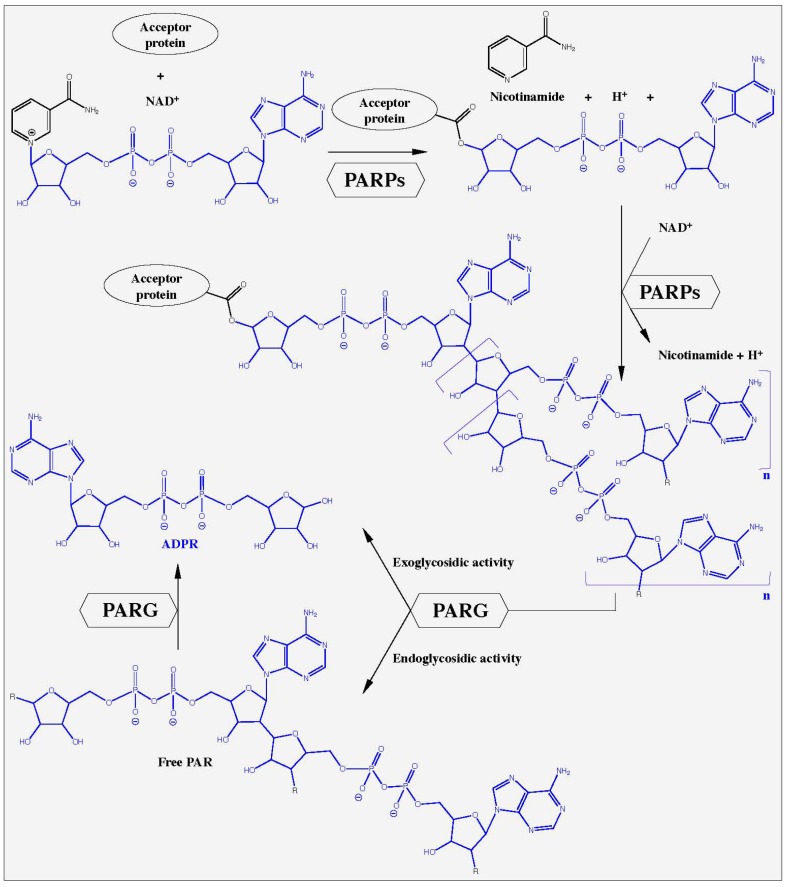
Poly(ADP-ribose) metabolism. In response to DNA damage, nuclear poly(ADP-ribose)polymerases (PARP-1 and PARP-2) hydrolyze NAD^+^, releasing ADPR, nicotinamide and H^+^. This reaction is induced by the formation of an ester bond between the amino acid-acceptors glutamic acid, aspartic acid or COOH-lysine and the first ADP-ribose (ADPR, marked in blue). Elongation occurs at the 2′-OH of the ribose moiety whereas branching occurs at the 2″-OH position resulting in poly(ADPR) (PAR), a multi-branched polyanion. Poly(ADPR)glycohydrolase (PARG) is a catabolic enzyme with an endo- and exoglycosidic activity. It cleaves the glycosidic bonds between the ADPR units, resulting in free PAR and finally ADPR.

The poly(ADP-ribosyl)ation of proteins is an early step in the initiation of DNA damage processing and cell death. The nuclear enzymes poly(ADP-ribose)polymerase-1 (PARP-1, EC 2.4.2.30, see [1] for nomenclature details) and to a lesser extent PARP-2 (EC 2.4.2.30) react to DNA nicks which occur after genotoxic stress within seconds, activating their catalytic activities and thereby producing large amounts of poly(ADP-ribose) (PAR) [2,3,4,5]. The substrate for this reaction is the respiratory coenzyme nicotinamide adenine dinucleotide (NAD^+^). The PARPs cleave NAD^+^ into ADP-ribose (ADPR), nicotinamide and H^+^, and catalyze the assembly of large negatively charged ADPR polymers with a branching frequency of up to 3% (Figure 1). The synthesis of PAR requires three distinct enzymatic activities: (1) initiation or mono(ADP-ribosyl)ation of the substrate, (2) formation of the PAR polymer, and (3) branching of the polymer. While over 90% of PAR is covalently bound to specific PARP enzymes, some PAR is linked to other proteins [3,6,7]. So far, the nuclear enzymes PARP-1 and PARP-2 are the sole members of the still growing family of PARP enzymes [6], whose catalytic activity is stimulated by DNA damage *in vitro* and *in vivo*, suggesting an integral role in the response to genotoxic insults [2,7,8,9,10]. About 99% of PAR is synthesized by PARP-1 after genotoxic stress demonstrating the dominant role of this enzyme in PAR metabolism [2,11].

The degradation of PAR is catalyzed mainly by PARG (PAR glycohydrolase, EC 3.2.1.143), an enzyme, with both exo- and endoglycosidic activities, hydrolyzing the glycosidic bond between ADPR units. PARG liberates primarily ADPR via exoglycosidic action, along with few free PAR chains from endoglycosidic action [12,13]. Branched and short PAR molecules are degraded slowly and with lower affinities by PARG (K_M_ ≈ 10 µM) than long and linear polymers (K_M_ = 0.1-0.4 µM) [14]. PARPs and PARG cooperate very closely in the response to DNA damage and imbalances between these enzyme activities dramatically alter the outcome of genotoxic stress, *i.e.* cell survival or death [3,6,12,15,16,17]. An overview of PAR metabolism is presented in Figure 1. The accumulation of PAR at sites of DNA damage recruits DNA damage repair proteins such as ATM, DNA ligase III, Ku70, MRE11, NBS1, and XRCC1 [18,19,20,21]. PAR also targets proteins involved in the epigenetic control of chromatin functions, such as histones, DNA methylation enzymes and transcription factors (reviewed in [22,23,24,25]). Recently, a distinct role for PAR, and its final cleavage product ADPR in Ca^2+^-mediated cell death has been elucidated [26,27,28,29,30].

The role of PARPs has been extensively studied in different genetic ablation models and more importantly, chemical inhibitors have found clinical acceptance recently [31,32,33,34,35,36]. In contrast, the detailed function of PARG remains still unclear. A single gene in mice and humans encodes PARG. Following transcription, several splicing products emerge that are translated into several proteins of different molecular sizes, subcellular localizations and the abilities to cleave PAR. In murine cells, the full-length 110 kDa PARG (mPARG110) is present in the cytoplasm and the nucleus and it accounts for most of the PARG activity. However, a shorter PARG protein (mPARG63) is described with ubiquitous distribution and mPARG58 localizes within the mitochondria [37,38,40]. In human cells, PARG exists at least in five different splicing variants [38,39,40]. Full-length hPARG111/110 is nuclear, two shorter isoenzymes hPARG103/102 and hPARG99 localize extra-nuclearly, and hPARG55 was found in mitochondria. Besides, hPARG60 has been shown in various localizations.

## 2. Lessons Learned from Genetic *parg* Knock-Out and Knock-Down Mice

The genetic modulation of PARG helped understand distinct biological functions of PARG isoforms. Interestingly, mice homozygous for complete knock-out of *parg* gene show an early embryonic lethal phenotype due to PAR accumulation [41]. Trophoblast stem cell lines derived from these *parg*-null embryos need the presence of PARP inhibitors to survive and proliferate. Moreover, these cells are hypersensitive to an alkylating agent and menadione. The genetic disruption of the *parg* gene enhances the level of PAR-modification of histones H1, H2A, and H2B, increases DNA accessibility in chromatin for MNase and acridine orange, and enhances DNA damage by *N*-methyl-*N’*-nitro-*N*-nitrosoguanidine (MNNG) and UV radiation [42]. These damage types remain unrepaired within 12 h. *Parg*-null trophoblasts show an increased level of cell death induced by the chemotherapeutic drugs epirubicin, cisplatin, and cyclophosphamide.

Depletion of the full-length nuclear form of the murine *parg* gene by deletion of exons 2 and 3 produces viable and fertile mice. The animals are characterized by a hypersensitivity to genotoxic stress and endotoxin-induced shock and are partially protected against renal and splanchnic ischemia/reperfusion damage (I/R) [37,43,44]. Embryo fibroblasts from these mice show hypersensitivity against genotoxic stress, develop more sister chromatid exchanges (SCE), contain more micronuclei and chromosomal aberrations and display abnormal centrosome amplification with a parallel accumulation of S-phase cells after aphidicolin-1 (Aph-1, replication poison) treatment compared with their wildtype counterparts. Moreover, PARG_110_^-/-^ cells accumulate more Rad51 foci in response to hydroxyurea. The observed defects in repair of replication fork damage could be the cause of higher rates of diethylnitrosamine-induced hepatocellular carcinoma in PARG_110_^-/-^ mice [45]. Following DNA damage by MNNG, PARG_110_^-/-^ cells display reduced formation of XRCC1 foci, delayed H2AX phosphorylation, reduced amounts of DNA break intermediates during repair, and an increased rate of cells undergoing cell death [46].

Two studies from Meyer-Ficca *et al.* [47,48] report that male PARG_110_^-/-^ mice are sub-fertile with abnormalities in nuclear condensation due to unusual removal of core histones, histone H1 linker-like nucleoproteins and TP2 prior to sperm maturation leading to abnormally shaped sperm nuclei with DNA strand breaks.

## 3. RNAi Technologies against PARG in Mammalian Cells

The use of RNAi approaches to abolish PARG protein in a variety of mammalian cells led to a diversity of results pending on cell type and stressor applied [15,16,17,30,49]. HeLa cells were transfected with sh-RNA against PARG [49]. This treatment increased radiosensitivity concomitant with defects in the repair of single- and double-strand DNA breaks. Irradiated PARG-deficient HeLa cells have abnormal centrosome amplification inducing either polyploidy or cell death by mitotic catastrophe. Plasmid based gene silencing of PARG in human A549 cells retarded the rate of single-strand break repair after H_2_O_2_ and reduced the number of surviving cells after a lethal application of this compound [15]. However, data from our group in murine embryonic fibroblasts (MEFs) using transient RNAi protocols demonstrate opposite results. PARG silencing was cytoprotective against H_2_O_2_ und could diminish cytotoxic Ca^2+^-influx mediated by TRPM2. This PARG-dependent cytosolic Ca^2+^ elevation was required for the translocation of AIF from mitochondria to the nucleus, a hallmark of PARP-1-dependent cell death [16,30] as described earlier [50]. Interestingly, RNAi against PARG could not prevent HeLa cells from alkylation-induced cell death [16].

## 4. The Use of Tannins as Inhibitors of PARG

**Figure 2:**
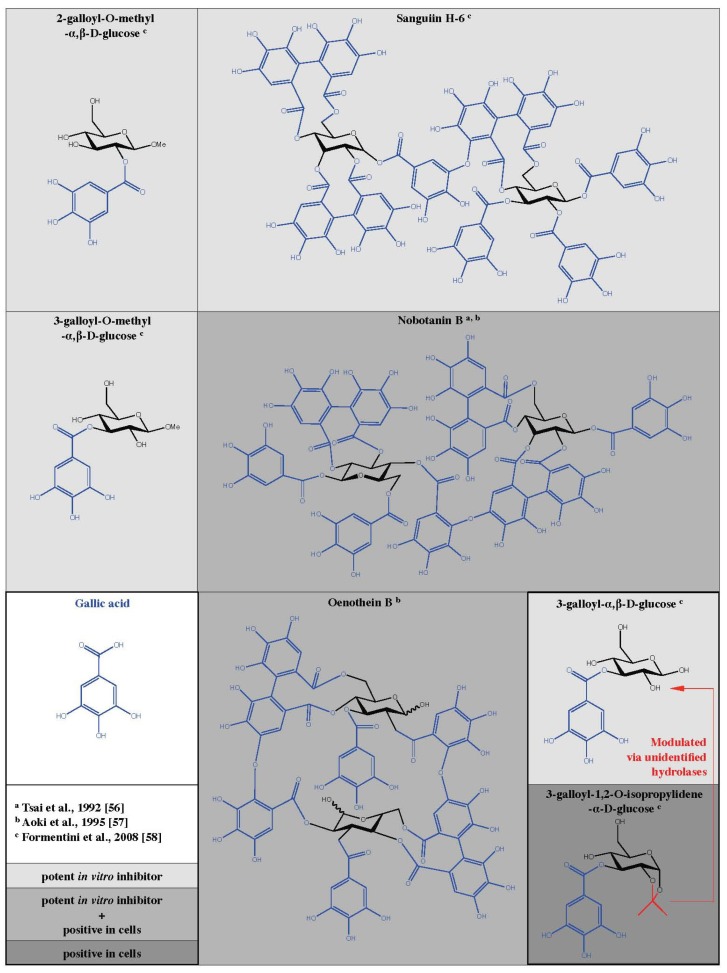
Chemical structures of galloyl derivates tested as PARG inhibitors with efficiency in cells and/or *in vitro*.

From various knock-out and knock-down models discussed above, the use of cell-permeable and specific PARG inhibitors would offer obvious advantages for research and therapeutic approaches. As early as 1989, Tanuma *et al.* described an inhibitory effect of tannin on purified PARG from human placenta [51]. The inhibition was dose-dependent and half maximal with 2.8 µg/mL. In the following section, the inhibitory actions observed with a variety of different tannin-like molecules are discussed. Hydrolysable tannins are commonly distributed in nature and represent multiple esters of gallic acid with glucose (Figure 2). At least 500 different glucogalloyl derivatives have been identified so far with molecular sizes varying between ca. 300 and about 10,000 Da [52,53].

### 4.1. Tannins Inhibit PARG in Vitro

All naturally existing tannins are a complex mixture of a variety of distinct molecules with a different chemical structure. Much of the work on tannins and PARG has been performed with the commercially available tannin cocktail gallotannin, isolated from oaks *(Quercus spec.)*. Some work has involved specific tannins from plants as different as *Thea sinensis* L. [54,55], *Saxifraga stolonifera* Meerb [54,55,56], *Geranium thunbergii* Sieg. et Zucc. [55,56], *Casuarina stricta* Ait. [55,56], *Cornus officinalis* Sieb. et Zucc. [55,56], *Rosa rogusa* Thunb. [55,56], *Coriaria japonica* A. Gray [55,56], *Tibouchina semidecandra* Cogn. [55,56,57], *Hetrocentron roseun* A. Br. et Bouch [55,56], and *Oenothera erythrosepala* Borbas. [55,57] indicating the widespread existence of tannins in the plant kingdom. The PARG *in vitro* inhibitory potential of tannins as well as related molecules is summarized in Table 1. 

Tsai *et al.* tested the inhibitory effects of tannins on a PARG preparation purified from human placenta that was characterized by a K_M_ value of 1.8 ± 0.3 µM and a V_max_ of 58 µMol/min/mg protein [54]. Using radiolabeled PAR as substrate, they determined the IC_50_ for ellagitannin (8.3-12.5 µM) and gallotannin (16.8-28.9 µM). Interestingly, they show that the degree of PARG inhibition increases with the number of galloyl groups, suggesting a specific structure-activity relationship. Furthermore, the authors showed that the conjugation of galloyl groups to glucose enhances PARG inhibition. The structurally related molecules (-)-epicatechin, (-)-epigallocatechin, and (-)-epigallocatechin gallate have little effect on PARG enzyme. Similar results were obtained with equivalent amounts of more complex tannins. Gallic acid, ellagic acid, benzoic acid and glucose could be excluded as PARG inhibitors even at concentrations of 100 µM [54]. In a follow up study, the inhibitory potential of tannins on PARG isolated from the nuclear fraction of murine mamma carcinoma cell line 34I (K_M_ = 2.1 ± 0.2 µM, V_max_ = 39 µmol/min/mg protein) was investigated [56]. Again gallic acid, ellagic acid, and glucose were inefficient as indicated by an IC_50_ value above 100 µM. The number of galloyl groups coupled to glucose seems a determinative factor. In fact, the IC_50_ values are decreasing for the tested compounds and range from 33.4 ± 3.1 µM for trigalloylglucose to 24.8 ± 0.2 µM for tetra-galloylglucose, and 17.8 ± 1.2 µM for pentagalloylglucose. The same rule was true if ellagitannins were studied. A higher level of molecular complexity promotes PARG inhibition. Monomeric ellagitannins are less efficient than dimeric or trimeric species. The most efficient tannin identified in this study is the tetrameric ellagitannin nobotanin K with an IC_50_ of 0.44 ± 0.03 µM. The dimeric ellagitannin nobotanin B (Figure 2) was almost ten times less effective *in vitro* (IC_50_ = 4.8 ± 0.4 µM) but developed a better PARG inhibitory potential when applied in cell culture compared with nobotanin K [56] (see next Section). Condensed tannins could not suppress PARG activity regardless of the complexity. The knowledge on PARG inhibiting tannins was extended by a study from Aoki *et al.* [57] using purified PARG from the nuclear fraction of calf thymus with a specific activity of 62 µmol/min/mg protein in an enzymatic assay with radiolabeled PAR as substrate. In this study, the half-maximal inhibition of PARG was determined at 15 µM of nobotanin B. In addition, they identified oenothein B (Figure 2), a compound isolated from vine plants, as a functional PARG suppressing molecule with an IC_50_ value of 3.8 µM acting in a cell culture model as well [57]. The PARG inhibitory potential of tannins and the structure-activity relationship discussed above were confirmed with purified PARG from the nuclear fraction of human placenta with a specific activity of 57 µmol/min/mg protein [55]. Trigalloyl-, tetragalloyl- and pentagalloylglucose show an increasing degree of PARG suppression depending on the number of galloyl groups. Condensed tannins are inactive, while ellagitannins inhibit PARG with an increasing efficacy depending on the molecular complexity. More recently, Keil *et al.* identified the PARG inhibiting properties of gallotannin *in vitro* by thin layer chromatography (TLC) of radiolabeled PAR together with nuclear extracts of HeLa S3 cells [66]. In this study, the gallotannin mixture contains molecules with 3-5 galloyl groups. 200 µM of gallotannin leads to a 40-fold elevation of PAR, indicating the PARG inhibitory effect. Moreover, this phenomenon was dose-dependent ranging between 50 and 400 µM of gallotannin. Similar results were shown by Falsig *et al.* using the TLC method as described above [66] but nuclear extracts were replaced by recombinant PARG (aa 378-976) [67]. As little as 5 µM gallotannin suppressed the majority of PARG enzymatic activity in this assay. To overcome the conglomerate nature of gallotannin, Formentini *et al.* separated all components of this tannin cocktail by high-performance liquid chromatography (HPLC) and clarified the chemical structure of 14 distinct tannin molecules by mass spectrometry (MS) techniques [58]. The authors found 4.2% of gallic acid in the gallotannin mix. Monomeric galloyl glucose derivatives were less prominent with values of 2.5% for mono-, 1.6% for tri-, and 3.1% for pentagalloyl glucose. In addition to tannins with only one glucose moiety, the dimeric sanguiin H-6 (contains two pentagalloylglucose subunits) was identified (Figure 2). Earlier it was shown that sanguiin H-6 is the main ellagitannin in fruits of red raspberries (*Rubus idaeus* L.) [68]. The PARG inhibiting properties of all identified galloyl derivatives were tested in an enzymatic assay with radiolabeled PAR as substrate [58]. The authors found that 10 µM of 3-galloyl-α,β-D-glucose, 3-galloyl-O-methyl-α,β-D-glucose, 2-galloyl-O-methyl-α,β-D-glucose as well as sanguiin H-6 were able to suppress about 65% of PARG activity *in vitro* (Figure 2). In contrast, the raw gallotannin mixture inhibits only 25% of PARG. For the three monomeric tannins evaluated positively, the IC_50_ values were determined: 0.95 ± 0.02 µM for 3-galloyl-α,β-D-glucose, 7.1 ± 0.05 µM for 3-galloyl-O-methyl-α,β-D-glucose, and 7.2 ± 0.03 µM for 2-galloyl-O-methyl-α,β-D-glucose. These results identify 3-galloyl-α,β-D-glucose as a very potent inhibitory molecule.

### 4.2. Tannins as PARG Inhibitors in Cells and Animals 

Before a detailed view of results of tannins as *in vivo* PARG inhibitors is discussed, it should be considered that the bioavailability of these compounds, *i.e.* solubility in culture medium and permeation into cells are major limiting factors for cell culture and animal use. Very little is known about the metabolic fate of tannins (for review see [69]). Gonzalez-Barrio *et al.* failed to detect either raspberry-derived compounds including sanguiin H-6 or any of their metabolites circulating in plasma of healthy humans after an acute consumption of 300 g raspberries within 24 h [70]. Likewise, only traces of ellagic acid (0.05%) and its metabolite ellagic acid-O-glucuronide (0.03%) were detected in urine from healthy volunteers 4-7 h after consumption. Interestingly, epicatechin derivatives were shown to reach the plasma of healthy volunteers and could be recovered as mother compounds together with their metabolites in the urine of rats and humans [53], ranging up to 31.4% in humans after consumption of 350 mL of a low-caloric, polyphenol-rich juice drink [71]. However, none of the catechin derivatives tested produced a significant PARG inhibition *in vitro* (see above). The degree of molecular complexity is considered as a major limiting factor for the bioavailability of tannins in the body [53]. None of the studies describing tannin derivatives as PARG inhibitors in cells and animals have evaluated the bioavailability of their test compounds. The only exception is a study performed by Tsai *et al.* [56]. They determined the bioavailability of nobotanin B in the murine mamma carcinoma cell line 34I and found that as little as 0.3% could be recovered 1 h after treatment with 30 µM nobotanin B in cell lysate.

#### 4.2.1. Tannins as PARG Inhibitors in Cell Cultures

A summary of results from cell culture experiments is presented in Table 2. Oligomeric ellgitannins were first tested in [56]. Nobotanin B, E, and K, originally identified as PARG inhibitors *in vitro*, were applied in a 34I cell line (mammarian carcinoma cells isolated from C3H mice). The authors labeled intracellular PAR by incorporation of [^3^H] adenosine (16 h) in the presence or absence of 10 µM of the test compounds for 1 h before challenging the cells with 100 nM dexamethasone with or without tannins respectively. Degradation of PAR attached to specific proteins (HMG 1, 2, 14, and 17 as well as histone H1) was monitored on acetic acid-urea-polyacrylamide-gels. Nobotanin B (Figure 2), but not E and K, inhibited the degradation of PAR under these conditions. Interestingly, this effect was dependent on the PAR acceptor protein. Whereas PAR degradation of modified HMG 14 and 17 as well as histone H1 could be inhibited in a dose-dependent manner by nobotanin B (3, 10 and 30 µM), this inhibition failed when PAR was bound to HMG 1 and 2. In addition, the glucocorticoid-dependent mRNA synthesis of MMTV (mouse mammary tumor virus) was analyzed in 34I cells 1 h after 100nM dexamethasone. In fact, nobotanin B was reducing MMTV mRNA synthesis in a range of 3, 10, and 30 µM. In a similar setting, oenothein B (Figure 2) was even more efficient than nobotanin B in a range of 1 to 50 µM of the tannins [57]. However, oenothein B (30 µM) inhibits the PAR degradation of PAR bound on HMG 14 and 17, but not when PAR is attached on the acceptor proteins HMG 1, 2, and histone H1. The concept of a negative effect of gallotannin on responsive elements was further investigated and confirmed on the 12-O-tetradecanoyl-phorbol-13-acetate (TPA)-induced HIV promoter activity in human leukemia T-cell line Jurkat [72,73]. In 2004 Uchiumi *et al.* [74], found that gallotannin responsive element(s) are located in the promoter region of the human *parg* gene of the human promyelocytic leukemia cell line HL-60.

Ying *et al.* investigated the effects of a crude gallotannin extract on cultured astrocytes isolated from ICR mice [75]. The cells were stressed with 300 µM of the oxidant H_2_O_2_ for 1 h to induce PARP-1-dependent cell death. Then the amount of cell death was quantified using the leakage of lactate dehydrogenase (LDH) into the culture medium. Gallotannin at concentrations of 100 nM up to 10 µM efficiently inhibited H_2_O_2_-mediated cell death at 24 and 72 h post challenge. Interestingly, this effect was only observed, when gallotannin was applied 1 h before the H_2_O_2_ treatment, but not when it was given concomitantly with H_2_O_2_. In cultures of neurons and astrocytes from Swiss-Webster mice, challenged with 100 µM H_2_O_2_ (1 h), neurons were protected from cell death when 1 µM nobotanin B or 100 µM gallotannin were administered 30 to 60 min to H_2_O_2_ [76]. The rate of cell death was assessed by propidium iodide (PI) staining of cell nuclei 20 to 24 h after the cytotoxic challenge. Concentrations above 10 µM nobotanin B and 100 µM gallotannin were neurotoxic *per se*. A neuroprotective effect of nobotanin B (10 µM) and gallotannin (50 µM) relative to *N*-methyl-D-aspartate (NMDA)-induced toxicity was discovered. Furthermore, astrocytes were treated with the oxidant H_2_O_2_ (300 µM, 1 h), the alkylating agent MNNG (100 µM, 5-60 min), and the peroxinitrite generator 3-morpholinosydnonimine (SIN-1, 5 mM, time not specified). Cell survival was measured by LDH content of cell lysates 24 h after the cytotoxic challenge. One hour pretreatment with either nobotanin B (0.2 and 2 µM) or 10 µM gallotannin protected against H_2_O_2_-induced astrocyte death. MNNG- and SIN-1-induced cell death could be suppressed with 50 µM gallotannin. As H_2_O_2_ and MNNG are PARP activating drugs, Ying *et al.* [76] determined the intracellular level of NAD^+^, the substrate for PAR synthesis, in astrocytes. After 10 min of 100 µM H_2_O_2_ or 100 µM MNNG, the drop of NAD^+^ levels was reduced by 50 µM gallotannin. Moreover, this reduced PAR degradation after 5 and 10 min of 100 µM MNNG as tested by PAR Western blot analysis. A similar result was obtained with H_2_O_2_ (data not shown). Finally, intracellular PAR accumulated in neuron/astrocyte co-cultures challenged with 300 µM MNNG for 15 min when 25 µM gallotannin was applied in parallel [76]. Taken together, these two publications by Ying *et al.*, [75,76] provide evidence that PARG inhibition by the tannins nobotanin B and the tannin raw extract gallotannin could have neuroprotective properties. However, the diversity of stressors, concentrations of chemicals, incubation times, cell lines and even assays, describing one and the same biological endpoint, presented in these publications preclude a final judgment.

In the year 2004, several publications appeared using gallotannin as PARG inhibitor in a variety of mammalian cells. Bakondi *et al.* investigated H_2_O_2_- and ONOO^-^-induced cell death in human adult (low calcium high temperature) keratinocytes (HaCaT) [77]. The cells were pretreated with 50 µM gallotannin for 30 min before a challenge with H_2_O_2_ (0.5 or 1 mM) or ONOO^-^ (250 or 500 µM). After 4 h (and 24 h, data not shown), cell death was quantified with the LDH leakage assay. Cells pretreated with gallotannin were less affected by cell death at 4 and 24 h. This observation was verified by the analysis of PI stained cell nuclei for the 4 h time point. Note that the extent of the cytotoxicity differed markedly between H_2_O_2_ and ONOO^-^. The chosen ONOO^-^ concentrations induced less than one third of cellular LDH release compared with the H_2_O_2_ conditions. Studying the mode of cell death, 300 and 500 µM H_2_O_2_, as well as 250 and 500 µM ONOO^-^ induced a caspase 3-like activity at about 6 h after the challenge. Interestingly, the values for ONOO^-^ treated cells were higher compared with the values of H_2_O_2_ treated cells. In all challenge conditions, a pretreatment with gallotannin (50 µM, 30 min) diminished the fluorescence signal indicative for caspase 3-like activity. Using immunocytochemistry, they found that gallotannin failed to block PAR accumulation after H_2_O_2_ and ONOO^-^. Moreover, gallotannin inhibited PARP in these settings as assessed by fluorescence microscopy of incorporated biotin-labeled NAD^+^. The authors confirmed their finding of a PARP inhibitory effect of gallotannin in an *in vitro* automodification assay with purified PARP-1 and increasing amounts of gallotannin. The drop of cellular NAD^+^ after H_2_O_2_ (500 µM) and ONOO^-^ (500 µM) treatment was analyzed 0.5, 1 and 2 h after the cytotoxic insult. Gallotannin could stabilize the cellular NAD^+^ pool at each time point and in both challenging conditions. However, in one of the two subsets of NAD^+^ assays presented in the publication, gallotannin alone increased the level of cellular NAD^+^ up to at least 150% after 2 h without any oxidative or nitrosative damage. From these data the authors conclude that “PARG inhibition by gallotannin maintains the inactive poly-ADP-ribosylated form of PARP and thereby breaks the poly(ADP-ribose) cycle” [77]. Di Meglio *et al.* investigated the effects of gallotannin (100 µM, 30 min pretreatment) on the NO donor spermine nonoate (SNO, 0.5 mM, 3 h) in rat germinal cells [78]. First, they could confirm the result from Bakondi *et al.* [77] that gallotannin (100 µM) inhibits PARP activity by about 60% reduced turnover of PARP-1 bound PAR (automodified/inactive form) in an *in vitro* assay. By single cell gel electrophoresis assay (COMET), gallotannin pretreatment prevented DNA repair in SNO challenged primary spermatocytes and round spermatids after a 2 h recovery period. The gallotannin-dependent defect in DNA repair capacity was accompanied by an elevated apoptotic rate as determined by terminal deoxynucleotidyl transferase dUTP nick end labeling (TUNEL) assay in spermatocytes and spermatids [78]. In another study addressing tannin interaction with DNA metabolism [82], mid-S phase cell nuclei of HeLa cells were analyzed *in vitro* for DNA replication activity. They found that in mid-S phase cell nuclei, the DNA replication was maintained during DNA repair. 30 µM of oenothein B (a potent PARG *in vitro* inhibitor, see above) blocked efficiently the DNA replication activity under these conditions. Recently, Tikoo *et al.* evaluated an effect of gallotannin on cell death induced by doxorubicin in H9c2 embryonic rat heart myoblasts [83]. In this study, the cells were treated with 1 or 5 µM doxorubicin for 24 h, then 25 µM gallotannin was applied for a subsequent time period of 24 h, and cell death was measured in LDH leakage assay, and the 3-(4,5-dimethylthiazol-2-yl)-2,5-diphenyltetrazolium bromide (MTT) metabolic cytotoxicity assay. Under all conditions tested, gallotannin treatment rescued almost 100% of the doxorubicin challenged cell population. This finding was supported by impaired cytoplasmic vacuolization, decreased bax expression, increased bcl-2 expression, and a reduced caspase-dependent cleavage of PARP-1, respectively. Interestingly, human breast cancer cells (MDA-MB-231) hyperreacted to gallotannin as it induced around 50% cell death (MTT) at a concentration of 25 µM. When MDA-MB-231 cells were challenged with doxorubicin (1 or 5 µM) prior to gallotannin administration, the presence of this tannin mixture elevated the cytotoxicity of doxorubicin [83]. These data demonstrate that a broad and even contrary spectrum of gallotannin-induced effects strongly depend on the cell line investigated. Rapizzi *et al.* evaluated the effect of gallotannin in murine macrophages (RAW 264.7) [80]. 30 µM gallotannin lead to an accumulation of PAR at 1 h without a toxic effect to the cells as assessed by biotinylated NAD^+^ incorporation, LDH, and MTT. In addition, they show a dose-dependent effect of gallotannin in stabilizing the decreased cellular NAD^+^ pool after 1 h of 100 µM MNNG. As a control, 100 µM gallotannin *per se* for 1 h had no influence on the basal NAD^+^ level. Another level of complexity was reached as they made the following interesting observation: gallotannin induced dose-dependent expression of iNOS and COX-2. Other tannins (ellagic acid, epicatechin gallate and gallic acid) and even more important RNAi against PARG failed to do so. IL-1β and TNFα were not affected by gallotannin. Consistent with a gallotanin/PAR-dependent transcriptional activation, elevations of iNOS and COX-2 were not related to the activation of NF-κB, AP-1, phospho-STAT-1 and IRF-1, nor mRNA stabilization [80]. This was the first evidence that pharmacological PARG inhibition by gallotannin alters PAR-dependent gene profiles. Erdelyi *et al.* investigated the effect of gallotannin (30 µM, 30 min pretreatment) on the expression of inflammation related proteins in the human lung adenocarcinoma epithelial cell line A549 challenged with either TNFα (20 ng/mL) or IL-1β (5 ng/mL) [81]. The treatment of gallotannin reduced the stress-induced expression (4 h after challenge) of the chemokines MCP-1, MIP-1β, RANTES, MCP-2, ENA-78, GCP-2, and fractalkine by at least 50%. Moreover, this reduction due to gallotannin was observed for the inflammatory cytokines IL-1α, IL-1β, IL-6, IL-8, and for the chemokine receptors CCR4 and CCR5, respectively. Interestingly, stress-induced MIP-3α and CXCR4 remained unaffected by gallotannin. All these data were collected from low-density arrays or PCR. Gallotannin reduced the phosphorylation of p38, ATF2 as well as ERK1/2 and CREB (Western blot). However, gallotannin *per se* induced the low-level phosphorylation of p38, ATF2, ERK1/2, and CREB. These findings were accompanied by a maximal phosphorylation of JNK and c-Jun. Surprisingly, an accumulation of PAR after TNFα or IL-1β in the presence of gallotannin was not observed. Therefore, the authors looked into the antioxidant properties of gallotannin in a peroxinitrite-driven DHR123 rhodamine converting assay (ABTS decolorization) and found that gallotannin is a strong antioxidant, explaining most of the observed results [81].

Another pharmacological effect of gallotannin in context with PARG suppression was observed in the modulation of a specific drug transporter type [79]. ATP-binding cassette (ABC) transporters are responsible for the transport of multiple substrates (e.g. drugs, metabolites, proteins) across cellular membranes. It has been shown that UVB irradiation inhibits ABC transporter in human peripheral blood mononuclear cells (PBMC) [79]. Dumitriu *et al.* provided evidence that the presence of gallotannin restored the UVB-induced ABC transporter inhibition. The authors concluded that both PARG and PARP-1 are necessary for the UVB-mediated ABC transporter suppression [79].

As it was obvious that the antioxidative potential of gallotannin has major impact on the outcome of experiments done in cells, effects depending on PARG inhibition had to be re-evaluated. Some work has been invested in clarifying this question [67,76,81]. As early as 1991, Ying *et al.* described that 10 µM of gallotannin reduced astrocyte death after 300 µM H_2_O_2_ (1 h). By comparison, 100-fold higher concentrations of the canonical antioxidants *N*-*tert*-butylphenylnitrone (PBN) and *N*-acetyl- cysteine (NAC) were required to match the gallotannin effect, indicating a minor involvement of the antioxidative properties of gallotannin in this experimental setting [76]. Falsig *et al.* invested an entire study on the relationship between PARG inhibition by gallotannin and benefits for the cell [67]. They used primary astrocytes from C57bl/6jbom mice and confirmed a cytoprotective effect of gallotannin against a series of stressors and incubation schemes (H_2_O_2_, MNNG, SIN-1). They could not observe an accumulation of PAR after gallotannin treatment in astrocytes challenged with 1 mM H_2_O_2_ for 1 h. Then, they investigated the nitric oxide scavenging potential of gallotannin in a cell-free assay and detected a highly efficient and dose-dependent reduction of NO production in the presence of gallotannin. The penetration of gallotannin across the membrane was assessed in the Caco-2 (human epithelial colorectal adenocarcinoma cell line) intestinal epithelial cell transport assay. No evidence was found that gallotannin would penetrate the cell monolayer (data not shown) [67]. Therefore, the authors attributed all beneficial effects of gallotannin observed in cell culture to the extracellular antioxidant effects of the tannin mixture.

In 2008, Formentini *et al.* took tannins back into account [58]. After the *in vitro* evaluation of distinct compounds isolated from the raw tannin extract gallotannin (see above section), they further tested the PARG inhibitory potential in HeLa cells challenged with 100 µM MNNG. Interestingly, they identified the highly hydrophilic molecule 3-galloyl-1,2-O-isopropylidene-α-D-glucose (Figure 2) as a potent inhibitor in cells. Whereas this tannin was inefficient *in vitro*, it was protective against MNNG-induced cytotoxicity depending on the treatment scheme as tested by LDH, and MTT assay. 3-galloyl-1,2-O-isopropylidene-α-D-glucose was able to inhibit PAR degradation induced by 100 µM MNNG as detected by Western blot and immunofluorescense microscopy techniques. Moreover a treatment of cells with 100 µM 3-galloyl-1,2-O-isopropylidene-α-D-glucose was sufficient to reduce MNNG-mediated translocation of AIF from mitochondria (4 h after treatment). AIF translocation from mitochondria to the cell nucleus was identified as a crucial step in PAR mediated cell death [16,30,50,84,85,86,87]. The application of this tannin-like compound did not induce DNA strand breaks (COMET assay) and had only slight cytotoxic effects pending on the dose and treatment scheme. Interestingly, 3-galloyl-1,2-O-isopropylidene-α-D-glucose was inefficient to inhibit PARG *in vitro*. Therefore, the authors suggested a metabolic activation step within the cell by hitherto unidentified hydrolases, generating the metabolite 3-galloyl-α,β-D-glucose, that was identified earlier as a very potent PARG inhibitor *in vitro* (Figure 2) [58].

#### 4.2.2. PARG Inhibitory Effects of Tannins in Animal Experiments

Since there is substantial doubt about the causality of a specific inhibition of PARG by tannin-like molecules in cell culture (see above), the following observations obtained in animal experiments should be considered with caution. A parenteral application mode for tannins in all animal studies was used [83,88,89,90,91]. Thus, it was possible to by-pass the low degree of oral bioavailability. The results of experiments with gallotannin as PARG inhibitor in animals are summarized in Table 3. In 2007 Wei *et al.* published a study with the raw tannin mixture gallotannin, performed in male Sprague-Dawley rats as a model of ischemia/reperfusion (I/R) injury [91]. The rats were subjected to intraluminal middle cerebral artery occlusion (MCAo) with subsequent reperfusion 2 h after the injury. They chose intranasal applications of gallotannin to ensure the direct by-pass of the blood-brain barrier. In the first set of experiments, they found that 50 mg/kg body weight gallotannin alters some physiological markers (data not shown), indicating a toxic potential of the drug when administered intranasally [91]. Therefore, they took a 25 mg/kg concentration, not altering body temperature, blood pressure, blood CO_2_ levels and pH. When gallotannin was applied 2 h after ischemia, they observed a protective effect of the drug mixture. 24 h after ischemia induction, the infarct size decreased nearly 70% in the brain and I/R-dependent neurological defects were reduced in gallotannin treated rats, respectively. These levels were still maintained 72 h after ischemia. In this setting, gallotannin increased nuclear PAR accumulation in the *penumbra* area of the brain 4 and 16 h after ischemia as assessed by immunofluorescence microscopy. Moreover, the translocation of AIF from mitochondria to cell nuclei − a marker for ongoing cell death − was prevented by gallotannin at 4 and 16 h post I/R damage. Even if gallotanin was applied intranasally 5 h post injury, infarct size and neurological defects were less abundant compared to control rats three days after I/R. Interestingly, the intravenous (*i.v.*) administration of 12.5 mg gallotannin did not result in any effects. The higher concentration of 25 mg/kg, that was efficient to reduce I/R-mediated insults when given intranasally, led to significantly increased death rate of the rats [91].

Tikoo *et al.* investigated the effects of gallotannin in a cisplatin-based model of nephrotoxicity in male Sprague-Dawley rats and came up with conflicting results pending on the time window of gallotannin administration [89].

The animals were treated with 5 mg/kg cisplatin intraperitoneally (*i.p.*) to induce nephropathologies. Gallotannin (10 mg/kg *i.p.*) was either applied directly or 3 days after cisplatin. A co-administration of gallotannin increased all markers of nephrotoxicity. The authors determined higher levels of blood urea nitrogen (BUN), plasma creatinine, and plasma albumin. Moreover, histopathological examinations revealed an increased tubular damage with a concomitant occurrence of caspase-dependent PARP-1 cleavage products as assessed on Western blot. Comparative studies with a reduced dose of cisplatin (1.5 µg/kg *i.p.*) developed almost similar nephrotoxicity in the presence of gallotannin. Interestingly, post-treatment of gallotannin (3 d after 5 mg/kg cisplatin *i.p.*) decreased nephrotoxicity as tested with determinations of BUN, plasma creatinine, plasma albumin, histopathologically evaluated tubular damage as well as a reduced amount of PARP-1 cleavage. It is noteworthy that gallotannin alone had no impact on body weight, BUN, creatinine, and albumin [89].

The effect of gallotanin on the antiretroviral drug azidothymidine [AZT, 28 d, daily 800 mg/kg *per os* (*p.o.*)] treated male Swiss albino mice was investigated by Tikoo *et al.* in 2008 [90]. As expected AZT administration led to severe hepatotoxic effects in mice. Gallotannin was given in parallel (28 d, daily 5 mg/kg *i.p.*) and showed hepatoprotective potential regarding AZT-induced liver insults. It normalized markers for oxidative stress in the liver [thiobarbituric acid reactive substances (TBARS), glutathione (GSH)]. In addition, the co-administration of gallotannin together with AZT resulted in a reduction of the following blood enzymes indicative for hepatotoxicity: alkaline phosphatase as well as the aminotransferases ALT and AST. AZT induces micronuclei formation in the peripheral blood due to its potential clastogenic and aneugenic activity. The administration of gallotannin decreased the number of micronuclei, respectively. Histopathologically, gallotannin increased the level of vacuolization, inflammatory infiltrations, and fatty degeneration in hepatocytes [90]. At the molecular level, gallotannin reduced the AZT-induced elevation of PARG protein in murine livers. This was determined by immunofluorescence stainings. As all antibodies against PARG developed so far failed to detect PARG protein in a cellular context, this result should be rated with caution. Furthermore, they observed a reduced portion of PARP-1 cleavage products in livers of gallotannin treated mice (Western blot) and a reduced amount of AZT-induced histone acetylation (data not shown).

A protective effect of gallotannin on doxorubicin-induced cardiotoxicity was observed in a breast cancer model in female Sprague-Dawley rats by Tikoo *et al.* [83] in 2010. Rats were treated with the carcinogen 7,12-dimethylbenz(a)anthracene (DMBA, 100 mg/kg *p.o.*) to develop a breast cancer phenotype. Then, the animals were exposed to doxorubicin (2.5 mg/kg, once per week for 5 weeks *i.p.*) with a subsequent administration of gallotannin (10 mg/kg, 5 d, daily, *i.p.*). As expected, animals challenged with doxorubicin showed a decline in cardiac systolic function and atrophy of the cardiac muscle. The application of gallotannin protected against doxorubicin-induced cardiotoxicity. The authors found improved heart weights, relative heart weights and decreased TBARS as marker for oxidative stress in the rat hearts. Moreover, gallotannin reduced the plasma level of LDH and partially restored myocardial morphology. At the molecular level, they observed that gallotannin reduced doxorubicin-induced bax expression in the hearts of the rats (Western blot) and a further increase of doxorubicin-induced p53 levels when the rats were treated subsequently with gallotannin [83]. However, gallotannin further potentiates the anti-cancer activity of doxorubicin in breast tumors, as assessed in the same animals.

Chandak *et al.* investigated male Sprague-Dawley rats in a streptozotocin (STZ)-induced model of diabetes type I (single *i.p.* injection of 55 mg/kg) [88]. After four weeks of STZ administration gallotannin was injected daily for four weeks (20 mg/kg *i.p.*) and clinical parameters were assessed. Gallotannin could significantly reduce the plasma creatinine level from 0.84 ± 0.10 mg/dl to 0.66 ± 0.44 mg/dl (around 0.4 mg/dL in control animals). The application of the tannin mixture had no effect on body weight, plasma glucose, BUN and urine protein in STZ-induced diabetes. By contrast, they found a marked reduction of glomerular hypertrophy by histopathology in gallotannin treated rats and a slightly reduced portion of the PARP-1 24 kDa cleavage fragment in the diabetic kidney [88].

## 5. Conclusions

The concept that tannin-like molecules could inhibit a key enzyme in DNA damage checkpoint signaling has attracted a lot of medical attention. The enzyme in question is PARG, which has been recently shown to play an integral role in the initiation of cell death. As reviewed here, some distinct compounds of the heterogeneous group of tannins have been identified as PARG inhibitors, but this action can only be observed *in vitro* and to a variable extent in cell cultures. The problem is that tannins have a limited bioavailability in biological systems due to their solubility and membrane permeation. Thus, the available literature on tannin actions in biological systems has to be interpreted with caution. Only few studies have verified the presence of PARG inhibitory principle within cells, either by analyzing the parent compound or a metabolite. Nevertheless, biological activity has been reported in both cells and animals. It is very likely that several of these actions are unrelated to PARG inhibition, but rather a consequence of the well documented antioxidative [52,53,69] or some other extracellular activities.

## Figures and Tables

**Table 1:** Inhibitory potential of tannins, related molecules as well as other known PARG inhibitors in enzymatic *in vitro* assays.

Compound				IC_50_ (µM)	Reference
**Hydrolyzable tannins**					
	Gallotannins				
		Trigalloylglucose		33.4 ± 3.1	[56]
				31.8 ± 2.8	[55]
			3-galloyl-α,β-D-glucose	0.95 ± 0.02	[58]
			3-galloyl-O-methyl-α,β-D-glucose	7.1 ± 0.05	[58]
			2-galloyl-O-methyl-α,β-D-glucose	7.2 ± 0.03	[58]
		Tetragalloylglucose		24.8 ± 2.0	[56]
				24.2 ± 1.9	[55]
		Pentagalloylglucose		17.8 ± 1.2	[56]
				18.9 ± 1.1	[55]
	Gallotannin mix			16.8 ± 28.9	[54]
	Ellagitannins				
		Monomer			
			Tellimagrandin I	10.8 ± 0.6	[56]
				11.9 ± 0.5	[55]
			Casuarictin	13.3 ± 0.7	[56]
				11.7 ± 0.6	[55]
			Geraniin	18.4 ± 0.7	[56]
				15.5 ± 0.6	[55]
		Dimer			
			Cornusiin A	5.1 ± 0.2	[56]
				7.1 ± 0.3	[55]
			Rugosin D	5.8 ± 0.3	[56]
				6.1 ± 0.4	[55]
			Coriariin A	8.1 ± 0.5	[56]
				8.5 ± 0.5	[55]
			Nobotanin B	4.8 ± 0.4	[56]
				4.4 ± 0.3	[55]
				15	[57]
			Oenothein B	4.8 ± 0.4	[55]
				3.8	[57]
		Trimer			
			Nobotanin E	1.4 ± 0.2	[56]
				1.8 ± 0.2	[55]
		Tetramer			
			Nobotanin K	0.44 ± 0.03	[56]
				0.38 ± 0.03	[55]
	Ellagitannin mix			8.3 ± 12.5	[54]
**Condensed tannins**					
	(-)-Epicatechin gallate				
		Monomer		>100	[56]
				>100	[55]
		Dimer		>100	[56]
				>100	[55]
		Trimer		>100	[56]
				>100	[55]
		Tetramer		>100	[56]
				>100	[55]
**Related compounds**					
Gallic acid				>100	[56]
Ellagic acid				>100	[56]
(-)-Epicatechin				>100	[55]
(-)-Epigallocatechin				>100	[55]
Benzoic acid				>100	[54]
Glucose				>100	[56]
**Other PARG inhibiting molecules**					
ADP-HPD				0.33	[59]
				0.136	[60]
				0.66	[61]
				0.12	[62]
*N-*bis-(3-phenylpropyl)-9-oxofluorene-2,7-diamide (GPI16552)				1.7	[63]
Eosin Y				1.9	[61]
GPI18214				3	[64]
Phloxine B				5	[61]
Ethacridine				7.2	[60]
Adenosine cyclic 3’, 5’-monophosphate				300	[65]
ADP-ribose				3200	[57]
				1100	[65]

**Table 2:** Tannin derivatives as PARG inhibitor in cell culture models.

Author	Cell system	Stressor	Compound	Effects
Tsai 1992 [56]	34I cells (C3H mouse mammarian carcinoma)	Dexamethasone	Nobotanin B	PAR-degradation of HMG 14, 17 and histone H1 blocked, but not on HMG1 and HMG2; Glucocorticoid-regulated MMTV (=Mouse mammary tumor virus) mRNA synthesis ↓; Bioavailability: 0.3%.
Aoki 1995 [57]	34I cells (C3H mouse mammarian carcinoma)	Dexamethasone	Nobotanin B/Oenothein B	Glucocorticoid-regulated MMTV mRNA synthesis ↓; Oenothein B → depoly(ADP-ribosylation of HMG 14 and 17 ↓ but not HMG 1, 2 and histone H1.
Ying 2000 [75]	Murine astro-cytes from ICR mice	H_2_O_2_	Gallotannin	Preincubated dose-dependent cell death ↓ (24 and 72 h); No effect under parallel treatment with H_2_O_2_.
Ying 2001 [76]	Neurons from Swiss-Webster mice	H_2_O_2_ H_2_O_2_, MNNG, SIN-1, NMDA	Nobotanin B/Gallotannin	Nobotanin B and gallotannin → H_2_O_2_- induced neuronal cell death (20-24 h) ↓; Nobotanin B and gallotannin → NMDA toxicity ↓; Glutamate toxicity ↓.
	Astrocytes from Swiss- Webster mice			Cell death ↓; Prevents NAD^+^ drop (10 min); PAR-degradation (5, 10min) ↓; Negligible anti-oxidative effect.
Bakondi 2004 [77]	HaCaT (Human kera- tinocytes)	H_2_O_2_, ONOO^-^	Gallotannin	Cytotoxic; Caspase 3-activity (6 h) ↓; No effect on PAR accumulation; PAR-PARP-1 automodification ↓; NAD^+^ drop after 0.5, 1 and 2 h ↓.
Di Meglio 2004 [78]	Rat germinal cells	SIN-1, SNO	Gallotannin	DNA repair ↓.
Dumitriu 2004 [79]	PBMC (Human peripheral blood mono-nuclear cells)	UVB	Gallotannin	Inhibition of ABC transporters following irradiation ↓.
Falsig 2004 [67]	Primary astro- cytes (C57bl/6jbom mice)	H_2_O_2_, SIN-1 MNNG CCM (=TNFα, IL-1β, IFN-γ)	Gallotannin	Cell death ↓; No effect on PAR. Cell death ↑ Gallotannin → nitrite formation from NO ↓; Gallotannin → iNOS ↑.
Keil 2004 [66]	HeLa (nuclear extracts)		Gallotannin	PAR levels *in vitro* (3 h) ↑; Degradation of PAR blocked.
Rapizzi 2004 [80]	RAW 264.7 (Murine macro- phages)	MNNG, LPS, IFN-γ, H_2_O_2_	Gallotannin	*Per se* iNOS ↑; COX-2 ↑; No induction of IL-1β and of TNFα; PAR accumulation (1 h) ↑ without any toxicity; No effect on basal NAD^+^ pool; NAD^+^ level stabilized after MNNG (1 h); No effect on AP-1, IRF-1 and pSTAT-1.
Uchiumi 2004 [74]	HL-60 (Human pro- myelocytic leu- kemia cells)	12-O-tetradeca- noylphorbol-13- acetate	Gallotannin	Nuclear PARG activity (3 h) ↓ but not cyto- plasmic; Basal relative PARG expression ↓.
Erdelyi 2005 [81]	A549 (Human lung adenocar- cinoma epithelial cells)	TNFα, IL-1β	Gallotannin	4 h after treatment: MCP-1 ↓; MIP-1β ↓; MCP-2 ↓; RANTES ↓; GCP-2 ↓; IL-6 ↓; ENA-78 ↓; fractalkine ↓; IL-1α ↓; IL-1β ↓; IL-8 ↓; CCR4 ↓; CCR5 ↓ by gallotannin, but not MIP-3α and CXCR4; Gallotannin *per se* induces IL-8; Binding of NF-κB to its consensus oligonucleotide ↓; NF-κB phosphorylation and nuclear translocation ↓; Gallotannin → basal and stress-induced AP-1 ↓; Gallotannin → pJNK ↑; p-p38 ↑; pATF2 ↑; pERK1/2 ↑; pCREB ↑; Protein phosphatases 1, 2A and PP1 ↓; No elevated PAR level. Gallotannin is *in vitro* a strong antioxidant.
Maruta 2007 [82]	Mid-S phase cell nuclei of HeLa cells		Oenothein B	DNA replication activity ↓.
Formentini 2008 [58]	HeLa (Human cervical cancer cells)	MNNG	3-galloyl-α,β-d-glucose	PAR degradation 15 and 30 min ↓; Cell death after 3 or 6 h ↓; AIF release ↓ (1 h, MNNG); No DNA strand break induction *per se*.
Tikoo 2010 [83]	H9c2 embryonic rat heart myoblasts	Doxorubicin	Gallotannin	Cell death ↓; Cytoplasmic vacuolization ↓; Bax ↓; Bcl-2 ↑; PARP-1 cleavage ↓.
	MDA-MB-231 breast cancer cells			Toxicity of doxorubicin ↑; Gallotannin is toxic *per se*.

**Table 3:** Effects of the potential PARG inhibitor gallotannin in animals.

Author	Animals	Model	Compound	Key findings
Tikoo 2007 [89]	Male Sprague-Dawley Rats	Model of nephrotoxicity (Cisplatin)	Gallotannin (intraperito-neal)	Co-treatment of gallotannin → nephrotoxicity ↑: (BUN ↑; Plasma creatinine ↑; Plasma albumin ↑); PARP-1 cleavage ↓; Tubular damage ↓. Post-treatment → toxicity ↓: (BUN ↓; Plasma creatinine ↓; Plasma albumin ↓); Tubular damage ↓; PARP-1 cleavage ↓.
Tikoo 2008 [90]	Male Swiss albino mice	Antiretroviral drug (Azidothymidine)	Gallotannin (intraperito-neal)	Gallotannin is hepatoprotective after azidothymidine. Oxidative stress ↓ (TBARS ↓, GSH ↓); ALT ↓; AST ↓; Alkaline phosphatase ↓; Micronuclei for- mation ↓; Vacuolization ↓; Fine inflammatory in- filtrations ↓; Fatty degeneration of hepatocytes ↓; Histone acetylation ↓; PARP-1 cleavage (89 kDa) ↓.
Wei 2007 [91]	Male Sprague-Dawley Rats	Model for ischemia/reperfusion injury (Intraluminal middle cerebral artery oc- clusion)	Gallotannin (intranasal)	Gallotannin protects against ischemia/reperfusion size ↓; Neurological deficits ↓; Nuclear PAR ↑ (4 and 16 h); AIF translocation ↓ (4 and 16 h). 5 h gallotannin after reperfusion: Infarct size ↓; Neurological deficits ↓.
Chandak 2009 [88]	Male Sprague- Dawley Rats	Model of diabetes I (Streptozotocin)	Gallotannin (intraperito-neal)	No effect on body weight, plasma glucose, BUN and urine proteine. Glomerular hypertrophy ↓; Plasma creatinine ↓; PARP-1 cleavage (24 kDa) ↓.
Tikoo 2010 [83]	Female Sprague- Dawley Rats	Breast cancer model (7,12-dimethyl benz(a)anthracene/ doxorubicin)	Gallotannin (intraperito- neal)	Gallotannin shows protection against doxorubicin- induced cardiotoxicity. Heart weight ↑; Relative heart weight ↑; TBARS ↑; Bax expression ↓; Plasma LDH ↓; Cytoplasmic vacuolization ↓; Myofibrils ↑; PARP-1 116 kDa + 89 kDa ↓; p53 ↑; But potentiates doxorubicin toxicity in breast tumors.
